# Hemichorea Induced by Non-ketotic Hyperglycemia in a Caucasian Woman

**DOI:** 10.7759/cureus.6866

**Published:** 2020-02-04

**Authors:** Kristina Pham, Sreenath Meegada, Tejo Challa, Prashanth Peddi, Madhavi Annakula

**Affiliations:** 1 Internal Medicine, Texas College of Osteopathic Medicine, Fort Worth, USA; 2 Internal Medicine, The University of Texas Health Science Center/Christus Good Shepherd Medical Center, Longview, USA

**Keywords:** hyperglycemia, hemichorea

## Abstract

Chorea is a disorder characterized by irregular, involuntary, hyperkinetic movements and has various causes. One unusual cause is hyperglycemia. This case involves a 76-year-old diabetic Caucasian female who developed gait disturbances, hemichorea of the face and limbs, and slurred speech over two to three weeks. She was found to have glucose level of 690 mg/dL with HbA1c of 14.7%. Head CT demonstrated hyperdensity in the left basal ganglia and mild involvement of right basal ganglia. Treatment with insulin alleviated her symptoms. The exact pathophysiology is unknown; however, many theories exist.

## Introduction

Chorea is a disorder characterized by irregular, involuntary, hyperkinetic movements [1]. This disorder can be inherited or acquired (autoimmune, cerebrovascular insults, drugs, infections, metabolic, toxic) and is associated with malfunctioning basal ganglia [1-2]. One unusual acquired cause of hemichorea, isolated movement disorder to one side of the body, is hyperglycemia [3]. The prevalence of this rare complication is unknown but it is most commonly seen in the elderly Asian women population [4]. Predisposing factors include poor glucose control, female gender, and advanced age [4-5]. The treatment for this disorder involves normalization of hyperglycemia, neuroleptics, antipsychotics, and anticonvulsants if chorea persists after correction of glucose [3]. With early diagnosis and treatment, this condition is reversible with good prognosis [5]. The best prevention is glycemic control.

## Case presentation

A 76-year-old Caucasian female with pertinent past medical history of type 2 diabetes mellitus, stroke, breast cancer, hypertension, and seizures presented to the ED with her family with complaints of gait disturbances for two to three weeks duration, right-sided hemichorea for four days duration, and right facial droop and slurred speech for one day duration.

The patient started falling down more frequently two to three weeks ago; however, the patient and her family denied any specific events that led to increased number of falls. Four days prior, the patient’s family noticed she was having uncontrollable right arm and leg movements. One day prior, the patient started having right facial droop and slurred speech. Her primary care provider was concerned for stroke and the patient was sent to the ED.

The patient denied prior episodes and stated this is the first time she is having these symptoms. She denied having pain with the movements and denied performing these movements on purpose. Uncontrollable movements do not change in amplitude or frequency with rest or with attempting purposeful movements. The patient reported having five strokes in her lifetime with the most recent one being 20 years ago; however, she was able to regain full function. She also reported having a seizure more than 20 years ago but could not recall any details. The patient denied being a current and former smoker, illicit drug use, and alcohol use.

On review of systems, the patient had pertinent positives of weight loss (20 pounds in the last three months), nausea, nonbloody vomiting, right-sided hemichorea, and gait disturbances (Video 1). She denied fever, headache, lightheadedness, changes in vision, chest pain, palpitations, shortness of breath, tingling, loss of sensation, polyuria, diaphoresis, anxiety, and depression.

**Video 1:** Right-sided hemichorea.

On physical examination, vital signs were within normal limits aside from BMI which was 28. She was in no acute distress and was alert and oriented. On neurological examination, the right pupil was not reactive to light but the left pupil was reactive to light. No consensual pupil light reflexes were present bilaterally. Extra ocular muscles were intact bilaterally with hyperkinesia of right side of face. All branches of cranial nerve 5 were intact: facial sensation equal bilaterally, jaw opening intact, and bite strength equal bilaterally. Cranial nerves 7, 8, 11, and 12 were intact. Upper and lower extremities sensation was intact and equal bilaterally. There was negative pronator drift, Babinski sign, and dysdiadochokinesis. There were +1 patellar reflexes bilaterally and +1 Achilles reflexes bilaterally. The finger to nose test demonstrated hyperdysmetria. On the musculoskeletal examination, the neck, bilateral upper extremities, and bilateral lower extremities had full range of motion and 5/5 strength. However, she had repeated circumduction motion of the right shoulder and repeated abduction and adduction of the right hip. 

At the time of admission, her blood glucose was 690 mg/dL with a normal anion-gap. Her glycosylated hemoglobin A1c was 14.7%. Her urinalysis showed glucose levels of 500 mg/dL and ketone levels of 15 mg/dL with pH of 5.5. There was also microscopic hematuria present. Other abnormal labs included: a white blood cell count of 3.3 x 103/microL and serum sodium of 125 mEq/L. Toxicology screen was negative.

**Figure 1:**
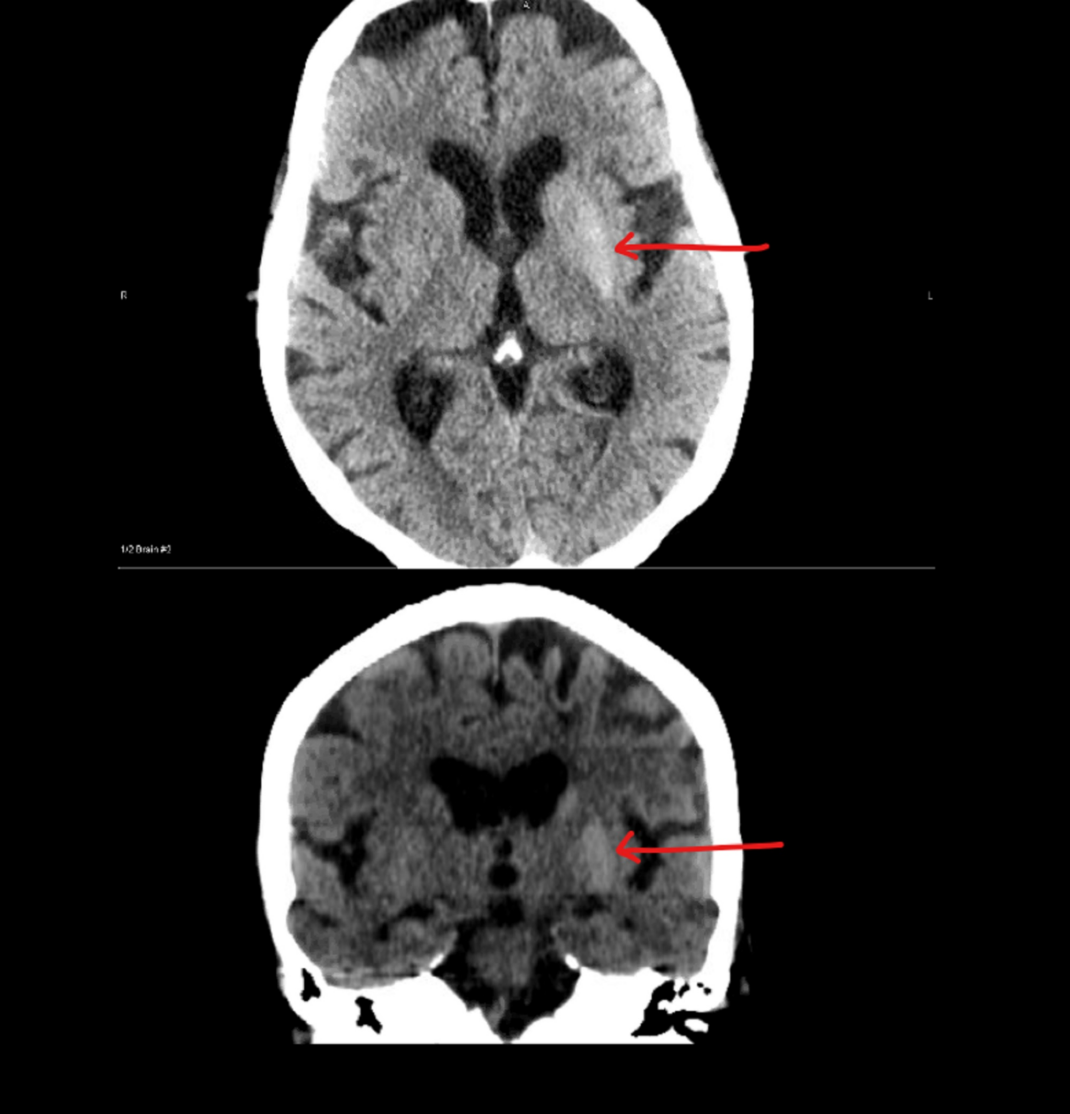
CT scan of head with out contrast showing asymmetric hyperdensity in the left basal ganglia.

No acute abnormalities were seen on chest X-ray and neck magnetic resonance angiography. Head CT scan showed atrophy, chronic small vessel disease, bilateral frontoparietal chronic encephalomalacia, and asymmetric hyperdensity in the left basal ganglia with possible mild involvement of right basal ganglia (Figure 1).

**Figure 2:**
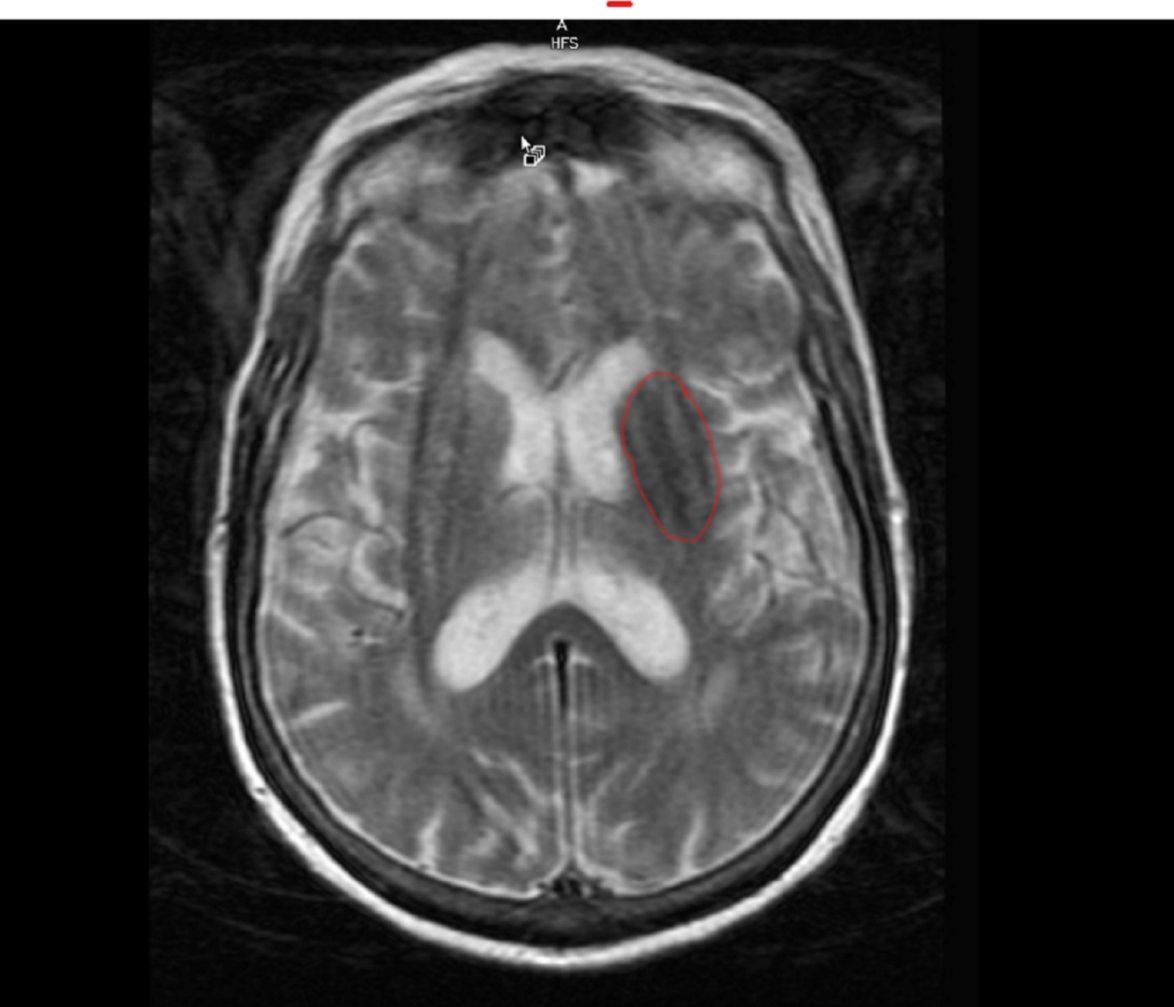
MRI brain T2 sequence showing hypodensity in left basal ganglia (area in red circle).

 MRI angiogram of brain without contrast showed no abrupt cut-off; however, scattered atherosclerotic plaques were present. MRI brain showed no acute stroke, moderate chronic white matter disease, and T2 sequence showed hypodensity in left basal ganglia (Figure 2).

The patient was treated with intravenous regular insulin and intravenous fluid hydration. Electrolyte abnormalities were corrected. The patient’s hemichorea gradually improved and she was discharged on the fifth day.

## Discussion

This is a report of a 76-year-old Caucasian female with pertinent past medical history of type 2 diabetes mellitus, stroke, breast cancer, hypertension, and seizures who developed gait disturbances, hemichorea of the face and limbs, and slurred speech over two to three weeks due to hyperglycemia. Her measured blood glucose was 690 mg/dL and glycosylated hemoglobin A1c was 14.7%. Head CT showed atrophy, chronic small vessel disease, bilateral frontoparietal chronic encephalomalacia, and asymmetric hyperdensity in the left basal ganglia with possible mild involvement of right basal ganglia. The patient’s symptoms gradually improved with treatment of intravenous regular insulin, intravenous fluid hydration, and electrolyte correction when present. This case report highlights typical and atypical findings in a rare presentation.

It has been published in the literature previously that up to 90% of hyperglycemic hemichorea reported cases have involved Asian patients [6]. It has also been hypothesized that elderly females are more predisposed to this condition due to postmenopausal changes in estrogen levels which affect interactions with gamma-aminobutyric acid and dopamine receptors in the nigrostriatal pathway and sub-thalamic nucleus [5-6]. However, other conflicting theories attribute this condition to abnormal perfusion, micro-hemorrhages, and mineral deposition in the basal ganglia [7]. 

While this patient fits the typical elderly female with poor glucose control, she is Caucasian instead of Asian. Some cases involving Caucasian patients have been described in the United States (US) and Europe [4-6]. With the increasing prevalence and incidence of type 2 diabetes mellitus in the US, there are a few points to consider [8]. It is important to realize that there may be an increase in the prevalence and incidence of hyperglycemic hemichorea in the near future as well. An overwhelming majority of cases have been reported in the Asian population leading to a hypothesis of a genetic predisposition; however, it would be not be surprising to see more cases in the American Indian, non-Hispanic black, and the Hispanic populations in the US as these populations have a higher prevalence of type 2 diabetes mellitus when compared with Asians [3, 8]. It is possible that many cases have been undiagnosed or misdiagnosed [6].

Hyperdensity in the basal ganglia contralateral to the hemichorea on her head CT is consistent with what has been reported in the literature. Brain MRI seems more commonly used which in those cases showed T1 putamen hyperintensity contralateral to the side of hemichorea or T2 hypodensity in the contralateral involved site [1, 4, 9].

The patient showed improvement of symptoms with treatment and this is consistent with the literature [1, 3-7, 9]. This patient was previously diagnosed with type 2 diabetes mellitus and presented with hemichorea due to chronically uncontrolled blood sugars, which is typical [6]. However, hemichorea can also present as an initial sign of uncontrolled glucose levels in an undiagnosed patient or in controlled disease [6].

## Conclusions

Patients with non-ketotic hyperglycemia (NKH) induced hemichorea have excellent prognosis. Hemichorea resolves in few days to few months after stabilizing blood sugar levels like our patient. NKH-related hemichorea can be the initial presentation of new onset diabetes type 2. Uncontrolled diabetes should be in the differential for patients presenting with new onset chorea or hemichorea. Our patient is unique because she is a Caucasian and has had absent light pupillary reflex in right eye in addition to NKH-induced hemichorea. To our knowledge similar case has not been reported in the literature.

For future studies, it would be interesting to see what factors would precipitate hemichorea at a specific moment in time when the patient has chronically uncontrolled diabetes for so long. There should also be more discussions on the role of past medical history and how much diseases such as hypertension, stroke, and seizure contribute to this condition.
